# Workshop report: INCF short course on neuroinformatics, neurogenomics, and brain disease, 14–21 September 2013

**DOI:** 10.3389/fnins.2015.00031

**Published:** 2015-02-26

**Authors:** J. Alexander Heimel, Rupert W. Overall, Robert W. Williams

**Affiliations:** ^1^Cortical Structure and Function Group, Netherlands Institute for NeuroscienceAmsterdam, Netherlands; ^2^Genomics of Regeneration, Center for Regenerative Therapies Dresden, Technische Universität DresdenDresden, Germany; ^3^Department of Genetics, Genomics and Informatics, Center for Integrative and Translational Genomics, University of Tennessee Health Science CenterMemphis, TN, USA

**Keywords:** short course, training, online resources, bioinformatic tools, data analysis

“To understand the brain and its disorders, we needed to get data. Now we have data, and we need to analyze it.”

On the 14th of September 2013, 37 scientists from all over the world assembled on the lovely Fraueninsel, in the Chiemsee lake in Bavaria. There they would stay for a week in the Frauenwörth Benedictine convent for the first Neuroinformatics Jamboree. Funded by the International Neuroinformatics Coordinating Facility and the University of Tennessee's Center for Integrative and Translational Genomics, the aim of the meeting was to introduce participants to core neuroinformatics and neurogenomics data sets and tools. On the program were a series of tutorials on these tools and resources and companion lectures by leaders in the field. The bulk of the program, however, was devoted to applying these tools and to learn how to mine the publicly available data in an ambitious attempt to assemble draft manuscripts by the end of the week.

The jamboree started with dinner and a welcoming speech by Sister Scholastica, head of the seminar program at the convent. In her entertaining manner, she informed the participants that, in fact, their goal was not only to achieve great science but, even more ambitiously, to work toward world peace by joining together with people from diverse cultures and backgrounds with the common aim of tackling problems in human health.

The next morning, the workshop proper began with a tutorial on systems neurogenetics online resources in general and GeneNetwork in particular. After breaking for lunch, research started. Participants assembled into groups with the titles “Neural Development,” “Addiction and Impulsivity,” “Brain Disorders,” “Synapse and Plasticity,” “Neurodegeneration,” and “Adult Neurogenesis.” These groupings were roughly based on common interests and assembled to have a mix of expertise and experience. Each group included at least one of the course lecturers to help catalyze progress. The afternoon was spent exploring possible research questions. With only one week to complete draft papers, it was essential that tractable questions were found quickly. After breaking for dinner, participants introduced themselves with a one-slide presentation about their background and research interests. After these introductions were over, more informal introductions ensued, and work on the research projects resumed. Most people chose to spend the evening working in groups to define good research questions. This general pattern was repeated for 5 of 7 days: lecture in the morning, lunch break, joint research, dinner, presentation, and more research.

By the afternoon of the second day, most groups had formulated research questions. These were presented to the other groups for open discussion. The topics varied not only in the types of questions posed, but also in the approaches suggested to attack them. Common in all topics, however, was the attempt to mine public data sets with online tools.

By the third day, all groups had really dug into their topic. The seven speakers (and occasionally also the participants) joined other groups to share their knowledge and expertise. In the afternoon, one participant spontaneously gave a tutorial on several tools (including Gemma and Cytoscape) that had not yet been covered. During that day, broadband access temporarily halted. Apparently, the Jamboree teams had already downloaded 30 GB of data—the abbey's whole allotment for the month of September! Fortunately, it was possible to increase the limit and by the end of the week close to 100 GB of data were sent to the island. By the fourth day, research had intensified, with only a single lecture in the morning. Groups were now really cracking, and the Jamboree was in full swing. Data were pouring in again, and the first lines of manuscripts were written using a simple template that was shared among the groups.

The fifth day was devoted to writing drafts. Documents were shared online via Dropbox or the very interactive Google Documents interface. People proofread and corrected each other's text while it was being typed. In the evening, the keyboards were temporarily left untouched while all teams presented the results of their labor. The morning of the sixth day was the deadline for submitting a first draft manuscript for open Jamboree review and editing. Participants were frantically trying to finish drafts, with all sections being edited simultaneously. By 1 p.m., manuscripts were submitted. Work was not over yet, as all these manuscripts were then reviewed by panels comprised of participants from the other groups. In a nice contrast to the usual review process, the reviewers were then on hand to orally explain their critiques and give constructive feedback on how to improve the drafts for publication. By 8 p.m., the scientific part of the Jamboree was over. Music, drinks, and a ping pong tournament finally managed to distract the participants from their research and celebrate the end of an intense, but marvelous week exploring the neuroscience and neurogenomics online databases and tools, and working toward world peace by creating lasting links with researchers from around the world.

The course was attended by 18 women and 19 men of 16 different nationalities working in 10 different countries, spanning the world from Australia to the USA, from Singapore to Portugal. All career stages were present: 5 professors, 10 scientists, 7 postdoctoral fellows, 14 PhD students and 1 practising psychiatrist.

By the end of the course, all six groups had produced a draft manuscript. The project drafts prepared during the workshop were then further worked on as long-distance collaborations. The resulting manuscripts passed peer review and can be found in the Research Topic accompanying this report.

More information, photographs, and the lecture slides are available at the jamboree website (https://sites.google.com/site/neuroinformaticsjamboree/).

## Conflict of interest statement

The authors declare that the research was conducted in the absence of any commercial or financial relationships that could be construed as a potential conflict of interest.

